# Using next-generation sequencing of microRNAs to identify host and/or pathogen nucleic acid signatures in samples from children with biliary atresia – a pilot study

**DOI:** 10.1099/acmi.0.000127

**Published:** 2020-06-12

**Authors:** Melvyn Smith, Mark Zuckerman, Apsara Kandanearatchi, Richard Thompson, Mark Davenport

**Affiliations:** ^1^​ Viapath Analytics, South London Specialist Virology Centre, Denmark Hill, London; ^2^​ Institute of Liver Studies and Paediatric Liver Services, Denmark Hill, London; ^3^​ Department of Paediatric Surgery, King's College Hospital NHS Foundation Trust, Denmark Hill, London SE5 9RS

**Keywords:** biliary atresia, next-generation sequencing, microRNA

## Abstract

Biliary atresia (BA) is a progressive disease affecting infants resulting in inflammatory obliteration and fibrosis of the extra- and intra-hepatic biliary tree. BA may be grouped into type 1 isolated; type 2 syndromic, where other congenital malformations may be present; type 3 cystic BA, where there is cyst formation within an otherwise obliterated biliary tree; and cytomegalovirus-associated BA. The cause of BA is unclear, with immune dysregulation, inflammation and infection, particularly with cytomegalovirus (CMV), all implicated. In this study a total of 50/67 samples were tested for CMV DNA using quantitative real-time PCR. Ten liver tissue and 8 bile samples from 10 patients representing the range of BA types were also analysed by next-generation sequencing. CMV DNA was found in 8/50 (16 %) patients and a total of 265 differentially expressed microRNAs were identified. No statistically significant differences between the various types of BA were found. However, differences were identified in the expression patterns of 110 microRNAs in bile and liver tissue samples (*P*<0.05). A small number of bacterial and viral sequences were found, although their relevance to BA remains to be determined. No direct evidence of viral causes of BA were found, although clear evidence of microRNAs associated with hepatocyte and cholangiocyte differentiation together with fibrosis and inflammation were identified. These include miR-30 and the miR-23 cluster (liver and bile duct development) and miR-29, miR-483, miR-181, miR-199 and miR-200 (inflammation and fibrosis).

## Introduction

Biliary atresia (BA) is a progressive disease affecting infants resulting in inflammatory obliteration and fibrosis of the extra- and intra-hepatic biliary tree. Worldwide incidence ranges from 1 : 50 00 to 1 : 20 000 live births, with 1 in 17 000 in the UK, equating to about 50 infants annually [1]. Surgical management involves carrying out a Kasai portoenterostomy, whereby the damaged extrahepatic biliary tree is removed in an attempt to restore bile flow from the native liver via residual bile ducts at the porta hepatis. This may initially be successful and clear the jaundice in 50–60 % of infants, although by adolescence, most affected patients require a liver transplant [2]. BA may be grouped by clinical phenotype into four variants: type 1 isolated, or perinatal BA (75–80 %); type 2 syndromic, or embryonic BA (7–10 %) with other congenital malformations, e.g. biliary atresia splenic malformation (BASM); type 3 cystic BA (5–8 %) with cyst formation within an otherwise obliterated biliary tree; and cytomegalovirus (CMV)-associated BA [3].

Most studies investigating an infectious cause of BA suggest an association with hepatotropic viruses, such as reovirus, rotavirus and, in particular, CMV [4, 5]. However, the relationship between CMV infection and BA is not conclusive; Fischler *et al*. [6] compared the impact of ongoing CMV infection at presentation of BA on the long-term outcome after the Kasai procedure. Twenty-eight patients with BA were investigated and no differences in survival with native liver or in survival after liver transplantation were found. Other viruses have also been implicated, including hepatitis B, hepatitis C, adenovirus, human papillomavirus (HPV), human herpes virus 6 (HHV6), Epstein–Barr virus (EBV) and BK virus [7–9]. These studies again are not conclusive and suggest that the presence of viruses may be a secondary phenomenon in BA. In addition to trying to identify an infectious cause of BA, a number of studies have implicated immune dysregulation as a pathogenic factor. There is also a significant amount of evidence that inflammation is a major factor [9–16].

In this study microRNA profiling using next-generation sequencing (NGS) was chosen as an unbiased approach to biomarker discovery [17, 18]. MicroRNA profiling was specifically selected, since it has a number of advantages: (i) the cell machinery required to enable posttranscriptional targeting by virally encoded or endogenous miRNAs is conserved in all eukaryotic cells, including hepatocytes [19]; (ii) miRNAs are associated with a number of human pathological conditions and are highly stable; (iii) they are produced by pathogens, including viruses, from which the genomic sequence signal has been lost, but due to the stability of miRNA, may be detected. It is also thought that while the biogenesis and mode of action of viral miRNAs may be similar to those of eukaryotic organisms, their target strategy may be different from that of their hosts. In this way viral miRNAs may have evolved to regulate a small number of host genes essential to their survival and if no viral target sequences can be found, it may still be possible to identify the virus by detecting the host response.

The aim of the study was to investigate whether an infection, immune dysregulation or other host genetic factors played a part in the development of BA in a paediatric cohort. Due to the lack of true non-BA controls, the isolated, idiopathic form was compared with samples representing BASM, choledochal malformations and CMV-associated BA.

## Methods

### Ethical statement

All samples used in the study were obtained from the Department’s BioBank with ethical approval, together with approval from the parents or guardians.

### Patients

In total, 230 samples from 67 children admitted with BA for a Kasai portoenterostomy procedure between 6 September 2010 and 11 July 2014 were collected (see Table 1). The median age was 66 days at sample collection (range 21–5763 days); 27/67 (40.3 %) were male. Each patient was given a unique study number.

**Table 1.** The type and number of samples collected during the study

No. of patients	34	5	3	21	4
**Sample type**	**Isolated BA**	**BASM**	**CMV-associated BA**	**Choledochal malformation**	Miscellaneous*
Blood	27	4	2	17	3
Bile	29	3	2	19	2
Liver	28	4	2	5	2
Biliary tract	0	0	0	1	0
Choledochal tissue	0	0	0	1	0
Urine	29	4	2	16	3
Stool	13	3	2	6	1
**Totals**	**126**	**18**	**10**	**65**	**11**

*Miscellaneous consisting of: PFIC (progressive familial intrahepatic cholestasis), gallstones, peroxisomal disorder, unknown liver disease.

### CMV DNA analysis

Samples were extracted using a QIAamp One-For-All Nucleic Acid kit on the Qiagen BioRobot MDx according to the manufacturer’s instructions (Qiagen, Manchester, UK). A total of 50/67 patient samples were tested for the presence of CMV DNA using the clinical laboratory diagnostic quantitative real-time PCR assay based on previously described methods [20, 21, modified with the inclusion of a feline herpesvirus extraction/inhibition control, added to the sample prior to extraction. Briefly, Qiagen Norox QuanitiTect Multiplex PCR Mix was used with 10 and 15 pmol of the CMV forward and reverse primers, together with 15 pmol of the FAM/BHQ1-labelled probe, together with 5 pmol of the forward and reverse primers and 1 pmol of the ROX/BHQ2-labelled feline herpesvirus probe in a 30 µl total reaction volume, which included 10 µl of the extracted sample. Reactions were run on a Rotor-Gene 6000 using the following cycling conditions: one cycle of 95 °C for 15 min, followed by 45 cycles of 95 °C for 15 s; 55 °C for 20 s (acquiring); and 65 °C for 20 s. Where there was sufficient volume, urine, whole blood or EDTA and bile samples were tested (see Table 2).

**Table 2.** CMV DNA PCR-positive samples

Patient code	BA classification	CMV DNA copies ml^−1^		CMV IgM status	Gender	Age at sampling (days)
		Urine	Bile			
64	CMV-associated	81 707	Not tested	Positive	M	84
3	CMV-associated	1 011 159	914	Positive	M	54
7	CMV-associated	Negative	176	Positive	M	67
13	CMV-associated	8387	Not tested	Positive	M	48
49	Isolated	10 376	Not tested	Negative	F	66
4	Isolated	1 424 882	552	Indeterminate	M	84
33	Choledochal malformation	220	Negative	Not tested	M	1650
38	Choledochal malformation	7620	Not tested	Not tested	F	1167

### Sequencing

Sequencing libraries were prepared from liver tissue and bile samples, matched where possible, from 10 patients. These consisted of 10 liver tissue and 8 bile samples from 5 isolated BA, 2 BASM, 2 choledochal cysts and 1 CMV-associated BA patient (see Table 3). RNA was extracted using Qiagen miRNeasy kits according to the manufacturer’s instructions; for liver tissues, 2×20 mg pieces were used, for bile 50 µl from each sample was used. Sequencing libraries were prepared using the Illumina TruSeq Small RNA kit (Cambridge, UK) according to the manufacturer’s instructions. Briefly, the concentration of RNA in each extract was assessed using a Qubit 2.0 fluorimeter (Thermo Fisher, Paisley, UK) and adjusted to 1 µg in 5 µl, 5′ and 3′adapters ligated as described and the index used for each sample recorded. Reverse transcription and amplification was carried out as recommended, using 6 µl of each adapter-ligated RNA library, but PCR cycling was adjusted from 11 to 15 cycles to increase yield. Bands (between 147–160 nt) from each sample were purified from 6 % agarose gels as described in the TruSeq Reference guide and analysed on the Qubit fluorimeter, and each indexed library was normalized to 4 nM and pooled. The indexed libraries (20pM) were analysed on a MiSeq instrument using a MiSeq Reagent kit v2 for 50 cycles, giving up to 26-cycle paired-end reads, with an average read length of 53 nt.

**Table 3.** Samples analysed by NGS, together with details of CMV status, gender and age at sampling

Patient code	Gender	BA classification	Sample type	Initial reads (B)	Initial reads (L)	Analysed for study (B/L)	Age at sampling (days)
3	M	CMV-associated	B and L	1 533 761	869 219	Yes/no	54
4	M	Isolated	B and L	1 134 645	331 748	Yes/yes	84
5	F	BASM	B and L	484 974	197 529	Yes/yes	79
6	M	BASM	B and L	430 307	455 692	Yes/yes	36
8	F	Isolated	B and L	162 633	162 633	Yes/yes	57
19	F	Isolated	B and L	2 002 992	2 457 683	Yes/no	110
26	M	Isolated	L	–	681 211	No/yes	44
27	F	Choledochal malformation	B and L	421 042	1 642 437	No/yes	113
39	F	Choledochal malformation	L	–	2 102 854	No/yes	94
42	F	Isolated	B and L	627 332	1 312 424	Yes/yes	54

B, bile fluid sample; L, liver sample.

Analyses were performed at the NIHR Biomedical Research Centre using the Translational Bioinformatics, Guy’s and St Thomas’ NHS Foundation Trust (London) using two investigative approaches. The adapter sequences were removed from all sequences in the fastq files using Trim Galore (v 0.3.7; https://www.bioinformatics.babraham.ac.uk/projects/trim_galore/) allowing 10 % mismatch and checked for quality with FastQC (0.11.2; https://www.bioinformatics.babraham.ac.uk/projects/fastqc/); sequences were analysed using miRBase (http://www.mirbase.org/index.shtml) and miRNA counts registered and the edgeR package (v 2.14; https://bioconductor.org/packages/release/bioc/html/edgeR.html) used for differential expression analyses based on the methods described by Wang *et al.* [22]. In each sample miRNA levels were quantified and normalized to account for the different total number of reads. For the second analysis all sequences were first compared with the human genome and the remaining unaligned sequences compared against a newly developed pathogenomic pipeline consisting of 2770 bacterial genomes and 5301 viral genomes assembled from the National Center for Biotechnology Information (NCBI) database. This was a package designed specifically for this project and was not developed further.

## Results

### Patients

Of the 67 children, 34 (50.7 %) presented with isolated BA, 5 (7.5 %) with BASM, 3 (4.5 %) with CMV-associated BA, 21 (31.3 %) with choledochal malformations and 4 (6.0 %) with a miscellaneous classification (see Table 1).

### CMV DNA loads and CMV IgM results

CMV DNA quantification was carried out in bile and urine samples collected from 50/67 patients. Evidence of CMV infection was found in eight (16 %) patients, three of whom were diagnosed with CMV-associated BA. Of the other five patients, three were diagnosed with isolated BA and two with choledochal malformations (see Table 2). CMV DNA was detected in the urine samples collected from two of the three patients with CMV-associated BA (3 and 64) and also in the bile of patients 3 and 7; there was insufficient bile sample from patient 64 for testing.

CMV IgM-positive results were noted in four of the six patients tested and one other was indeterminate. Two of the patients with CMV IgM-positive and indeterminate results had isolated BA. Most patients had been referred from other hospitals and for 30 of 67 there were no CMV serology results available when requested. Further samples had not been tested as a baseline. However, CMV DNA was negative in the urine samples from 17 of these patients, therefore excluding active CMV infection at diagnosis. Of the rest, there were no CMV results for any samples available.

### Sequencing

Table 3 shows details of the 18 bile and liver tissue samples from 10 patients (4 male and 6 female), together with their biliary atresia classification and initial number of reads. After adapter removal, the general pattern across all samples was of three main sequence peaks of 22 nt (corresponding to the expected miRNA length), 32 nt and one of 50 nt. However, 3 samples (bile 27, liver 3 and 19) showed very low numbers of reads (fewer than 1000 matches against known miRNAs) and were excluded from further analyses. This resulted in a total of 15 samples for analysis.

### MicroRNA expression in all samples of the four biliary pathologies

**Fig. 1.**
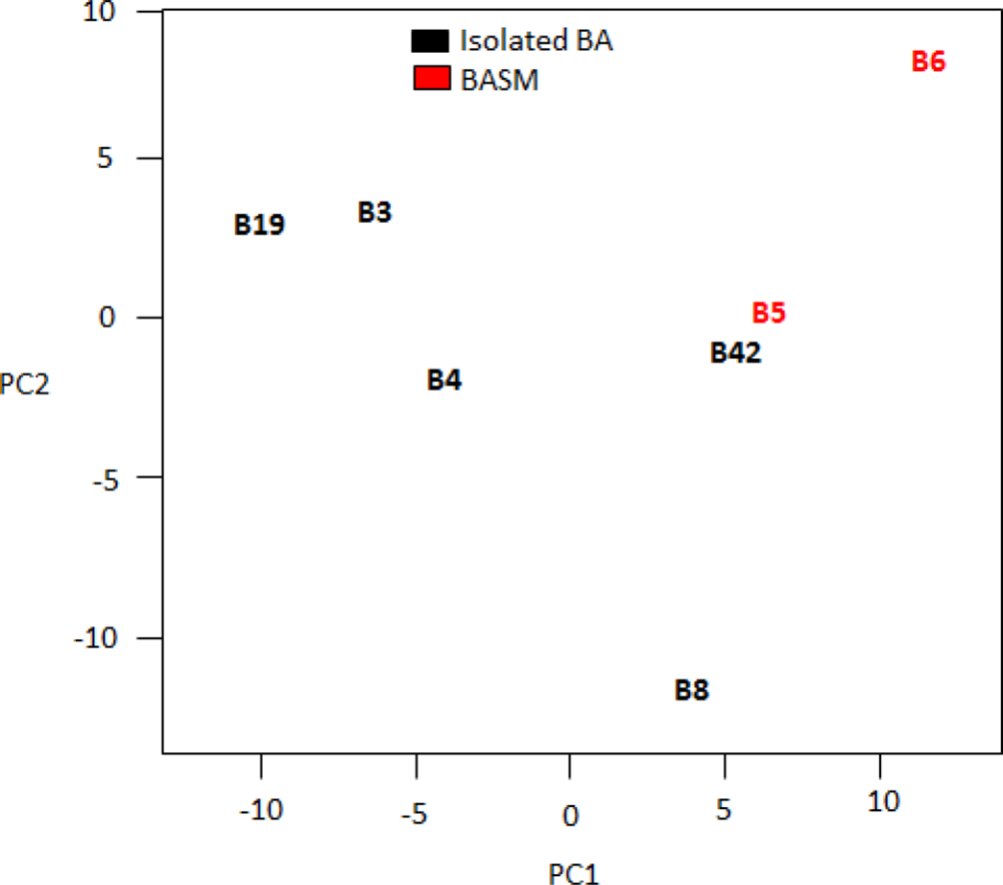
Principal component analysis on the log of normalized counts for the bile fluid samples.

**Fig. 2.**
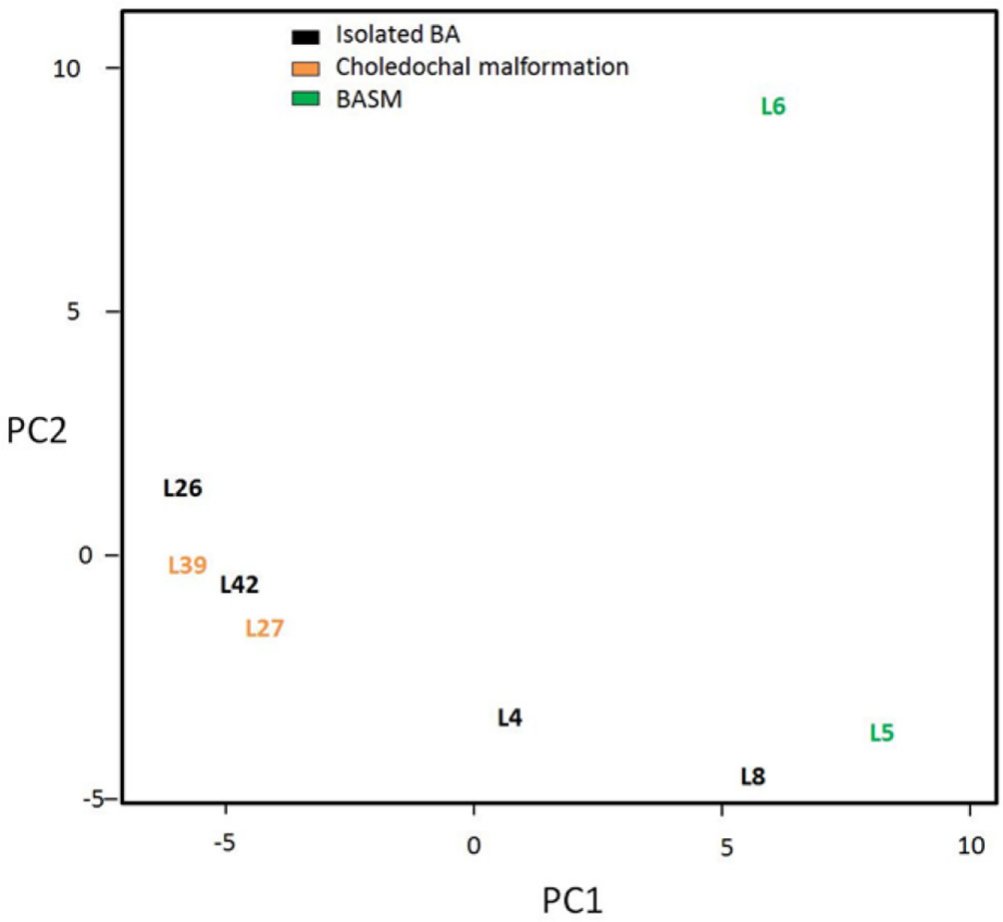
Principal component analysis on the log of normalized counts for the liver tissue samples.

A total of 265 differentially expressed miRNAs in bile and liver samples were found (Table S1 and Fig. S1, available in the online version of this article). In order to determine if there were any significant differences in microRNA expression patterns between the different forms of BA, principal component analysis was performed on the sequence data from bile and liver samples separately (see Figs 1 and 2).

No statistically significantly differences between the different forms of BA were found, that is the type 1, idiopathic forms displayed similar microRNA expression patterns to the known (choledochal malformation, BASM and the CMV-associated) forms. The PCAs do suggest a difference between the expression patterns of bile and liver tissues from the two BASM samples (5 and 6) and the other types of BA, although the sample numbers are too small to form a definite conclusion. There were, however, statistically significant differences overall between miRNA expression between bile and liver samples for all pathologies.

Preliminary analysis of both bile and liver samples failed to show the presence of any viral or bacterial microRNA sequences. A small number of bacterial genomic sequences, including *Propionibacterium propionicum* (in liver samples) and *Enterococcus mundtii* in bile samples were noted. However, it is extremely unlikely that these have any significance for BA.

### Pathway analysis for microRNAs involved in fibrosis

**Fig. 3.**
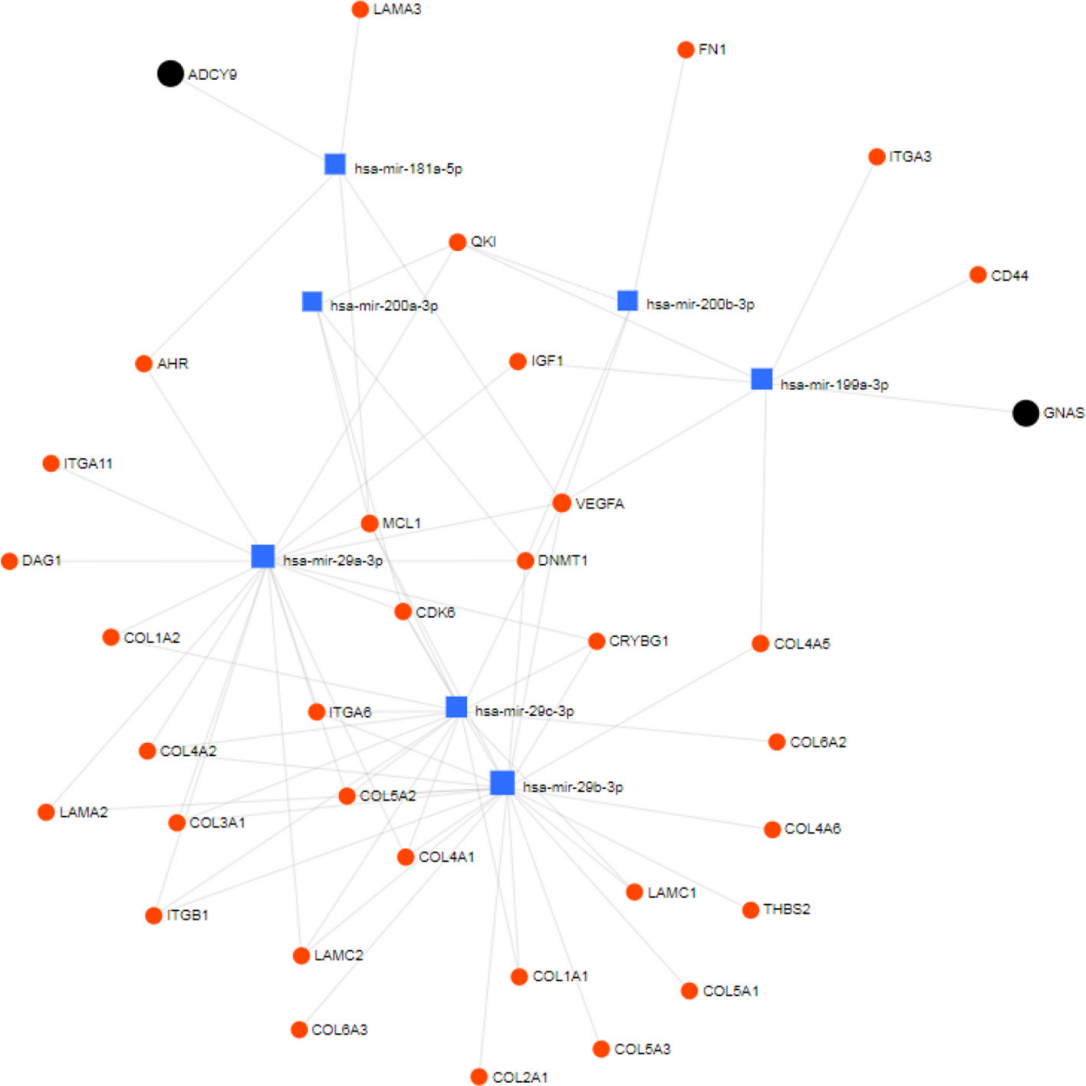
MicroRNA pathway interaction network showing microRNAs involved in inflammation reviewed in the Discussion. The pathway was constructed with miRNet (accessed on 1 July 2019) using the KEGG database and highlighting genes involved in ECM receptor interactions (as red dots) and bile secretions (as black dots) for liver tissue; microRNAs are shown as blue squares. There were no annotations in the KEGG database for mir-23 or mir-483.

A pathway analysis was carried out for a number of microRNAs mentioned in the literature associated with fibrosis using miRNet (https://www.mirnet.ca/). These include: mir-29a-3p; mir-30 and the mir-23 cluster; mir-29b-3p; mir-29c-3p; mir-181a-5p; mir-199a-3p; mir-199b-3p; mir-200a-3p; mir-200b-3p; mir-483-3p; and mir-483-5p. The pathway was constructed using the KEGG database and highlighting genes involved in ECM receptor interactions and bile secretions for liver tissue. There were no annotations in the KEGG database for the mir-23 cluster, mir-199b-3p or mir-483, (see Fig. 3). Table 4 includes a brief description of the gene functions taken from GeneCards (https://www.genecards.org/).

**Table 4.** Details of the genes involved in the network shown in Fig. 3

Gene	Name	Function
ADCY9	Adenylate cyclase type 9	Catalyses the formation of cyclic AMP from ATP – regulated by a family of G protein-coupled receptors
GNAS	Guanine nucleotide-binding protein (G protein), alpha stimulating	G proteins function as transducers in numerous signalling pathways controlled by G protein-coupled receptors
AHR	Aryl hydrocarbon receptor	Cell cycle regulation – plays a role in the development and maturation of many tissues
CD44	Haematopoietic cell E- and l-selectin ligand	Involved in cell–cell interactions, cell adhesion and migration – interacts with other ligands, e.g. collagens and matrix metalloproteinases
CDK6	Cyclin-dependent kinase 6	A serine/threonine protein kinase, important for cell cycle G1 progression and G1/S transition
COL1A1	Collagen type 1 alpha 1 chain	Encoding the pro-alpha chains of type I collagen
COL1A2	Collagen type 1 alpha 2 chain	
COL2A1	Collagen type 2 alpha 1 chain	Encoding the alpha-1 chain of type II collagen (forms part of the ECM in liver)
COL3A1	Collagen type 3 alpha 1 chain	Encoding the pro-alpha1 chains of type III collagen
COL4A1	Collagen type 4 alpha 1 chain	Integral component of basement membranes – interacts with other ECM components, e.g. perlecans, proteoglycans and laminins
COL4A2	Collagen type 4 alpha 2 chain	One of the six subunits of type IV collagen, the major structural component of basement membranes
COL4A5	Collagen type 4 alpha 5 chain	One of the six subunits of type IV collagen, the major structural component of basement membranes
COL4A6	Collagen type 4 alpha 6 chain	
COL5A1	Collagen type 5 alpha 1 chain	Encoding the alpha chains for the low-abundance fibrillar collagen type V
COL5A2	Collagen type 5 alpha 2 Chain	
COL5A3	Collagen type 5 alpha 3 Chain	
COL6A2	Collagen type 6 alpha 2 chain	Encoding the alpha chains of type 6 collagen found in most connective tissues – important in organizing matrix components
COL6A3	Collagen type 6 Alpha 3 chain	
CRYBG1	Crystallin beta-gamma domain containing 1	Gene ontology (GO) annotations related to this gene include carbohydrate binding – may exert its effects through interactions with the cytoskeleton
DAG1	Dystroglycan 1	A central component of dystrophin–glycoprotein complex-related pathways, including VEGF signalling
DNMT1	DNA methyltransferase 1	Transferring methyl groups to cytosine nucleotides of genomic DNA
FN1	Fibronectin 1	Cell adhesion and migration processes, including embryogenesis, wound healing, blood coagulation and host defence
IGF1	Insulin-like growth factor 1	Mediating growth and development
ITGA11	Integrin subunit alpha 11	Associated with collagen binding and collagen receptor activity and may be involved in attaching muscle tissue to the extracellular matrix
ITGA3	Integrin subunit alpha 3	Joins with a beta 1 subunit – interacts with extracellular matrix proteins, including members of the laminin family
ITGA6	Integrin subunit alpha 6	May associate with a beta 1 or beta 4 subunits – interacts with extracellular matrix proteins, including members of the laminin family
ITGB1	Integrin subunit beta 1	Cell adhesion and recognition in a variety of processes e.g. embryogenesis, haemostasis, tissue repair and immune response
LAMA2	Laminin subunit alpha 2	Component of the basement membrane organizing cells into tissues during embryonic development by interacting with other extracellular matrix components
LAMA3	Laminin subunit alpha 2	Essential for formation and function of the basement membrane and has additional functions in regulating cell migration and mechanical signal transduction
LAMC1	Laminin subunit gamma 1	An extracellular matrix glycoprotein and a non-collagenous constituent of basement membranes. They have been implicated in a wide variety of biological processes, including cell adhesion, differentiation, migration and signalling
LAMC2	Laminin subunit gamma 2	A non-collagenous constituent of basement membranes – implicated in a wide variety of biological processes e.g. cell adhesion, differentiation, migration and signalling
MCL1	MCL1 apoptosis regulator, BCL2 family member	Regulation of apoptosis – mediates its effects by interactions with a number of other regulators of apoptosis
QK1	KH domain containing RNA binding	RNA-binding protein regulating pre-mRNA splicing, export of mRNAs from the nucleus, protein translation and mRNA stability
THBS2	Thrombospondin 2	A disulphide-linked homotrimeric glycoprotein -–mediates cell-to-cell and cell-to-matrix interactions
VEGFA	Vascular endothelial growth factor A	Active in angiogenesis, vasculogenesis and endothelial cell growth, induces endothelial cell proliferation, promotes cell migration and inhibits apoptosis

### Differences in microRNA expression between bile fluid and liver tissue samples

**Fig. 4.**
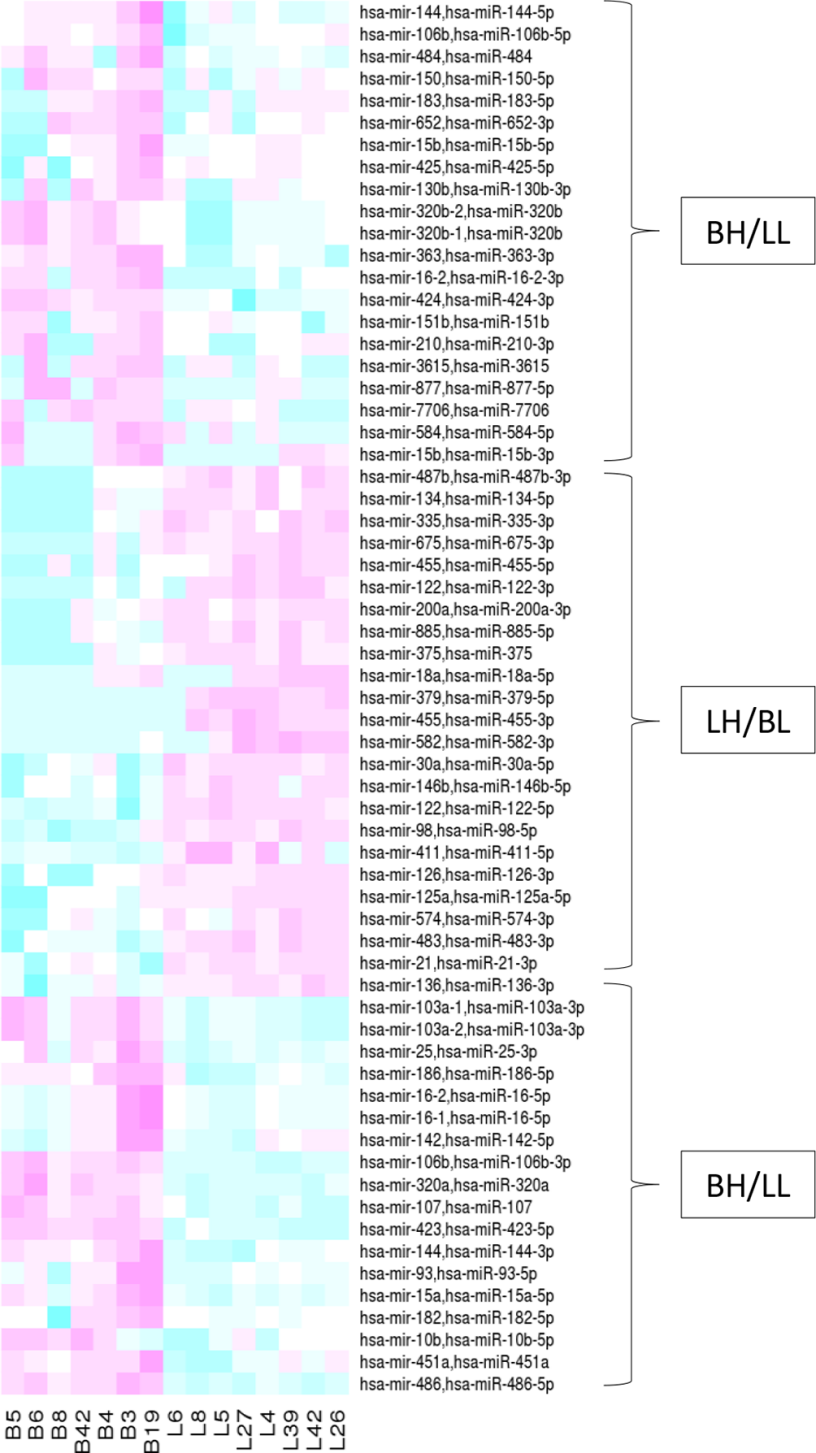
miRNAs significantly differentially expressed between bile and liver tissue samples (*P*=<0.05). Each column represents a sample, every row a miRNA. Pink represents high levels of expression and blue low levels of expression. Results are scaled for each row and are therefore only comparable between samples. The expression patterns can be broadly dived into two groups: bile high, liver low (BH/LL) and liver high, bile low (LH/BL).

A further analysis of the differences in microRNA expression patterns associated with inflammation and fibrosis between liver and bile samples is shown in Tables 5 and 6 for liver and bile, respectively. The average fold changes for all 265 microRNAs from bile fluid and liver tissue samples were calculated and compared. After removing duplicate mature microRNAs with identical data, this resulted in the identification of 110 microRNAs with statistically significant (*P*<0.05) patterns of differential regulation between liver tissue and bile samples, (see Fig. 4, Table 7).

**Table 5.** miRNAs associated with fibrosis found in liver samples

miRNA name	L26 (isolated)	L27 (chol. cyst)	L39 (chol. cyst)	L4 (isolated)	L42 (isolated)	L5 (BASM)	L6 (BASM)	L8 (isolated)
hsa-miR-29a-3p	1212.61	638.74	1488.56	835.3	739.55	160.12	473.4	338.36
hsa-miR-29b-3p	169.2	124.2	368.14	50.62	102.4	80.06	0	33.84
hsa-miR-29c-3p	84.6	70.97	192.07	50.62	91.02	0	0	0
hsa-miR-30a-3p	338.4	159.68	448.17	101.25	193.42	0	52.6	101.51
hsa-miR-30a-5p	8065.24	8534.26	7971.01	6353.34	5438.53	6965.06	10940.76	4398.71
hsa-miR-30b-5p	564	1135.54	832.31	582.18	568.88	600.44	420.8	778.23
hsa-miR-30c-2-3p	28.2	35.49	16.01	25.31	11.38	0	0	0
hsa-miR-30c-5p	648.6	762.94	1072.41	658.12	523.37	880.64	420.8	1082.76
hsa-miR-30d-3p	0	17.74	32.01	50.62	0	0	0	33.84
hsa-miR-30d-5p	3243.02	3814.69	5282	6226.78	2821.67	6684.86	8678.97	6428.89
hsa-miR-30e-3p	56.4	425.83	624.24	227.81	238.93	240.17	105.2	169.18
hsa-miR-30e-5p	3186.62	2377.53	4369.65	5669.92	3071.97	2882.1	6522.38	3315.95
Has-miR-122-5p	7444.84	24981.78	19511.37	25793.06	15075.43	47754.72	18094.34	30960.17
hsa-miR-140-3p	705	550.03	864.33	354.37	523.37	280.2	315.6	203.02
hsa-miR-140-5p	28.2	35.49	16.01	25.31	22.76	0	0	0
hsa-miR-143-3p	104002.13	76755.11	99941.75	45334.02	42063.3	67248.89	57859.81	45780.44
hsa-miR-181a-5p	11674.86	18239.54	11348.29	23388.41	10820.18	28380.63	34715.88	26256.93
hsa-miR-181b-5p	761.4	727.45	752.28	885.92	455.11	840.61	999.4	1522.63
hsa-miR-199a-3p	8206.24	2661.41	5986.26	3974	4721.74	2882.1	4628.78	3823.5
hsa-miR-199a-5p	1325.41	621	1184.45	759.36	978.48	200.15	631.2	744.4
hsa-miR-199b-3p	8206.24	2661.41	5986.26	3974	4721.74	2882.1	4628.78	3823.5
hsa-miR-199b-5p	507.6	195.17	176.07	101.25	261.69	40.03	368.2	169.18
hsa-miR-200a-3p	479.4	248.4	272.1	126.56	261.69	80.06	368.2	473.71
hsa-miR-200b-3p	310.2	17.74	208.08	177.18	102.4	40.03	263	203.02
hsa-miR-483-3p	2171.41	3743.72	5666.14	1265.61	1592.88	1721.25	789	1894.83
hsa-miR-483-5p	141	124.2	240.09	151.87	68.27	160.12	210.4	304.53

**Table 6.** miRNAs associated with fibrosis found in bile samples

miRNA name	B19 (isolated)	B3 (CMV-associated)	B4 (isolated)	B42 (isolated)	B5 (BASM)	B6 (BASM)	B8 (isolated)
hsa-miR-29a-3p	807.87	111.22	259.97	292.51	0	0	0
hsa-miR-29b-3p	90.77	30.9	0	0	0	0	0
hsa-miR-29c-3p	308.62	117.4	0	97.5	0	0	0
hsa-miR-30a-3p	45.39	6.18	279.97	97.5	0	0	236.69
hsa-miR-30a-5p	925.87	302.77	4319.55	1755.04	295.94	682.5	2603.56
hsa-miR-30b-5p	671.71	605.55	59.99	97.5	147.97	0	0
hsa-miR-30c-2-3p	0	0	20	0	0	0	236.69
hsa-miR-30c-5p	2105.91	741.49	79.99	0	0	0	355.03
hsa-miR-30d-3p	27.23	49.43	59.99	0	147.97	0	0
hsa-miR-30d-5p	4220.9	3744.52	2619.73	1755.04	1183.76	2957.5	1065.09
hsa-miR-30e-3p	190.62	129.76	439.95	0	0	0	710.06
hsa-miR-30e-5p	3059.02	2551.96	939.9	292.51	443.91	0	236.69
hsa-miR-122-3p	27.23	0	20	0	0	0	0
hsa-miR-122-5p	1207.27	12.36	1439.85	682.52	591.88	227.5	591.72
hsa-miR-140-3p	889.57	858.89	499.95	682.52	147.97	0	236.69
hsa-miR-140-5p	36.31	18.54	20	0	0	0	0
hsa-miR-181a-5p	37643.15	32841.76	34576.42	60 744	46906.49	87587.42	47574.08
hsa-miR-181b-5p	1062.03	630.27	1519.84	1267.53	1923.61	7279.99	2366.87
hsa-miR-199a-3p	5782.18	1550.95	12058.75	10140.25	8582.26	455	16449.74
hsa-miR-199a-5p	726.18	105.04	679.93	585.01	0	0	710.06
hsa-miR-199b-3p	5782.18	1550.95	12058.75	10140.25	8582.26	455	16449.74
hsa-miR-199b-5p	453.86	61.79	399.96	195	147.97	0	1183.43
hsa-miR-200a-3p	199.7	37.07	20	97.5	0	0	0
hsa-miR-200b-3p	172.47	6.18	20	0	0	0	0
hsa-miR-483-3p	45.39	6.18	79.99	97.5	0	227.5	118.34
hsa-miR-483-5p	54.46	0	159.98	0	295.94	0	0
hsa-miR-675-3p	18.15	0	20	0	0	0	0

**Table 7.** Showing the difference in expression between the average of all the bile and liver tissue samples where statistical significance was reached (*P*>0.05), together with the log_10_ counts per million (c.p.m.) and false discovery rates (FDRs)

Mature	logFC	logc.p.m.	*P* value	FDR
hsa-let-7d-5p	2.41	9.58	0.01741	0.04840
hsa-let-7i-5p	1.46	13.27	0.01343	0.03953
hsa-miR-103a-3p	2.49	12.19	2.47E−05	0.00025
hsa-miR-106b-3p	4.39	11.64	1.72E−15	1.52E−13
hsa-miR-106b-5p	3.23	8.77	0.00050	0.00282
hsa-miR-107	3.36	11.62	5.48E−09	1.45E−07
hsa-miR-10b-5p	1.01	15.87	5.66E−05	0.00047
hsa-miR-122–3 p	−5.25	7.72	2.96E−06	4.13E−05
hsa-miR-122–5 p	−5.09	13.67	3.84E−13	2.55E−11
hsa-miR-125a-5p	−2.09	10.05	0.00028	0.00178
hsa-miR-126-3p	−2.22	10.66	3.98E−05	0.00035
hsa-miR-126-5p	−0.62	11.96	0.01910	0.05166
hsa-miR-128-3p	2.70	7.52	0.04767	0.11179
hsa-miR-128-3p	2.85	7.47	0.03431	0.08464
hsa-miR-130b-3p	4.06	7.49	3.10E−05	0.00030
hsa-miR-134-5p	−2.72	5.76	0.00120	0.00577
hsa-miR-136-3p	−3.07	9.37	1.31E−05	0.00015
hsa-miR-139-5p	−3.21	5.75	0.02101	0.05514
hsa-miR-142-5p	3.46	11.94	0.00094	0.00482
hsa-miR-143-3p	−2.31	15.57	0.03022	0.07699
hsa-miR-144-3p	5.67	11.83	8.37E−09	1.85E−07
hsa-miR-144-5p	7.11	11.37	8.26E−09	1.85E−07
hsa-miR-145-5p	−3.61	8.75	0.00314	0.01243
hsa-miR-146b-3p	−2.20	6.99	0.01225	0.03775
hsa-miR-146b-5p	−2.42	11.91	0.00018	0.00116
hsa-miR-148a-3p	−2.70	14.78	0.01043	0.03369
hsa-miR-148b-3p	−1.67	8.48	0.00327	0.01274
hsa-miR-150-5p	2.30	7.90	0.00039	0.00240
hsa-miR-151a-5p	2.45	10.83	0.01166	0.03678
hsa-miR-151b	3.52	7.38	0.00050	0.00282
hsa-miR-154-5p	−3.39	4.33	0.03863	0.09306
hsa-miR-15a-5p	5.71	11.34	4.43E−10	1.47E−08
hsa-miR-15b-3p	3.47	6.41	0.00095	0.00482
hsa-miR-15b-5p	4.25	8.94	6.35E−05	0.00051
hsa-miR-16-2-3p	7.32	9.59	1.85E−11	9.79E−10
hsa-miR-16-5p	6.11	14.80	1.81E−08	3.43E−07
hsa-miR-17-3p	3.93	6.50	0.00262	0.01067
hsa-miR-181a-5p	1.08	15.06	0.00866	0.02981
hsa-miR-181b-5p	0.46	10.49	0.01324	0.03953
hsa-miR-181b-5p	0.50	10.47	0.01197	0.03733
hsa-miR-182-5p	5.11	11.89	3.26E−07	4.80E−06
hsa-miR-183-5p	4.02	8.06	0.00023	0.00152
hsa-miR-185-5p	3.06	6.34	0.00400	0.01513
hsa-miR-186-5p	2.32	11.66	0.00078	0.00429
hsa-miR-18a-5p	−2.74	7.81	0.00208	0.00876
hsa-miR-191-5p	2.06	12.54	0.02081	0.05513
hsa-miR-192-5p	−1.02	15.56	0.00895	0.03003
hsa-miR-193b-3p	−2.65	6.92	0.03073	0.07756
hsa-miR-200a-3p	−1.70	7.61	0.00201	0.00872
hsa-miR-200b-3p	−1.31	6.83	0.01614	0.04598
hsa-miR-203a	−2.61	5.56	0.03450	0.08464
hsa-miR-204-5p	−2.20	8.20	0.04815	0.11193
hsa-miR-210-3p	3.69	7.77	1.01E−05	0.00013
hsa-miR-21-3p	−4.90	11.13	2.91E−07	4.53E−06
hsa-miR-23b-3p	−1.22	8.79	0.03628	0.08821
hsa-miR-24-3p	−1.53	8.27	0.01772	0.04840
hsa-miR-25-3p	3.28	12.96	6.99E−06	9.27E−05
hsa-miR-26a-5p	−0.93	14.39	0.00848	0.02969
hsa-miR-27b-3p	−1.09	14.64	0.01340	0.03953
hsa-miR-27b-5p	−2.12	4.71	0.01769	0.04840
hsa-miR-29a-3p	−1.24	9.03	0.01308	0.03953
hsa-miR-29b-3p	−1.74	6.46	0.00986	0.03227
hsa-miR-29b-3p	−1.71	6.44	0.01154	0.03678
hsa-miR-301a-3p	2.36	7.29	0.01501	0.04359
hsa-miR-30a-5p	−2.59	12.19	0.00014	0.00098
hsa-miR-30b-5p	−0.51	8.98	0.02159	0.05610
hsa-miR-30d-5p	−0.41	11.99	0.03228	0.08070
hsa-miR-30e-5p	−0.69	11.37	0.00262	0.01067
hsa-miR-320a	2.79	10.59	2.16E−09	6.37E−08
hsa-miR-320b	3.30	8.06	1.80E−07	3.10E−06
hsa-miR-331-3p	−2.55	5.01	0.00534	0.01940
hsa-miR-335-3p	−2.28	6.39	0.00124	0.00586
hsa-miR-33b-5p	2.86	6.72	0.01513	0.04359
hsa-miR-3615	4.45	6.63	3.49E−05	0.00033
hsa-miR-363-3p	6.41	9.17	1.64E−10	6.20E−09
hsa-miR-375	−3.36	7.99	6.57E−05	0.00051
hsa-miR-376c-3p	−4.38	5.38	0.00422	0.01574
hsa-miR-379-5p	−6.10	4.66	0.00142	0.00659
hsa-miR-411-5p	−2.73	9.57	8.26E−05	0.00060
hsa-miR-421	2.71	6.83	0.00789	0.02827
hsa-miR-423-3p	0.57	9.34	0.04126	0.09851
hsa-miR-423-5p	6.18	12.98	3.29E−19	4.36E−17
hsa-miR-424-3p	2.78	7.05	2.20E−05	0.00024
hsa-miR-424-5p	−2.53	8.84	0.00288	0.01157
hsa-miR-425-5p	3.57	8.95	0.00168	0.00755
hsa-miR-429	−1.60	5.80	0.04652	0.11007
hsa-miR-451a	3.78	15.24	1.27E−05	0.00015
hsa-miR-455-3p	−6.10	4.74	0.00206	0.00876
hsa-miR-455-5p	−3.35	7.35	0.00146	0.00668
hsa-miR-483-3p	−6.14	10.39	8.79E−11	3.88E−09
hsa-miR-484	2.66	8.55	0.00104	0.00522
hsa-miR-485-3p	−3.86	4.95	0.00918	0.03041
hsa-miR-486-5p	7.48	18.35	1.25E−21	3.31E−19
hsa-miR-487b-3p	−2.89	6.13	0.00041	0.00246
hsa-miR-532-5p	0.84	8.95	0.02346	0.06036
hsa-miR-542-3p	−3.14	7.18	0.00440	0.01620
hsa-miR-574-3p	−3.22	8.78	0.00081	0.00438
hsa-miR-574-5p	0.80	6.22	0.04947	0.11301
hsa-miR-582-3p	−5.86	6.74	4.77E−05	0.00041
hsa-miR-584-5p	5.47	7.07	0.00016	0.00110
hsa-miR-589-5p	2.93	5.81	0.02001	0.05357
hsa-miR-652-3p	3.88	6.96	8.41E−05	0.00060
hsa-miR-660-5p	0.55	9.00	0.04870	0.11222
hsa-miR-675-3p	−2.89	5.38	0.00187	0.00825
hsa-miR-744-5p	2.46	6.66	0.00351	0.01346
hsa-miR-7706	3.02	5.62	0.00093	0.00482
hsa-miR-877-5p	3.18	5.04	0.00115	0.00564
hsa-miR-885-5p	−3.61	7.07	0.00047	0.00274
hsa-miR-93-5p	3.47	10.62	7.38E−05	0.00056
hsa-miR-98-5p	−1.91	10.58	4.01E−05	0.00035

## Discussion

We used NGS to determine the miRNA profiles in liver tissue and bile samples from 10 patients in a paediatric cohort of 67 children with BA to determine whether there were different expression patterns between type 1 (unknown) and the known congenital forms of biliary disease (choledochal malformation and BASM) and CMV-associated BA. As liver and bile samples are unavailable from healthy donors, those from the congenital and CMV-associated conditions were used as controls to determine any differences in expression unique to type 1BA.

We focused on examining clinical pathology samples rather than using the more commonly examined cell culture and animal model systems in the hope of finding more direct evidence for the involvement of human- and pathogen-derived miRNAs. Overall, CMV infection was detected in eight (16 %) patients, three of whom were diagnosed with CMV-associated BA. Of the other five patients, three were diagnosed with isolated BA and two with choledochal malformations. It was challenging drawing together CMV serological and molecular results involving different sample types with the groupings, especially as, for example, two of the patients with CMV IgM-positive and indeterminate results had isolated BA. Most patients had been referred from other hospitals and there were no CMV serology results available when requested for 30 of 67. However, CMV DNA was negative in the urine samples from 17 of these patients, therefore excluding active CMV infection at diagnosis. Of the rest, there were no CMV results for any samples available, making any further interpretation difficult other than from the findings of other diagnostic tests carried out. Overall, it could be concluded that as CMV is such a ubiquitous infection, it is likely that there will be a group of children with BA in whom it is difficult to elucidate the pathogenesis in the presence of more than one possible aetiology.

We were particularly interested to see if there was a distinct pattern of expression in the CMV-associated form of biliary atresia and also in finding clear evidence that delineated type 1 BA. Liver tissue and bile samples were analysed to maximize the discovery potential of the investigation. Although the extrahepatic biliary system is involved in the early stages of BA, the intrahepatic bile ducts are subsequently involved in the majority of patients [23]. As the disease progresses, the fibrous obliteration of the bile ducts causes the bile to become trapped and it is likely that the resulting damage to hepatocytes and cholangiocytes contributes to the miRNA patterns found in the bile fluid. Thus by examining bile fluid and liver tissues, a more accurate profile of miRNA expression relating to BA may be found.

A broad overview of the statistically significant differences in microRNA expression is shown in the heat map (Fig. 4). This highlights the clear differences between bile and liver tissue samples and separates the expression patterns into two main groups: (i) high levels of expression in bile, low in liver and (ii) high levels of expression in liver, low in bile. The heatmap (Fig. S1) shows the expression patterns of all 265 microRNAs found in all samples. Here again broad patterns of expression can be seen, with an intermediate pattern, a low-level expression pattern and high levels of expression for both bile and liver samples. In the following sections some of the more intensively studied microRNAs that were also found in our work are briefly discussed in the context of the published literature.

### MicroRNA expression in liver samples

### MicroRNA involvement in liver development

#### Mir-30

Hand *et al*., [24] identified upregulation of the miR-30 family during the late stages of murine foetal development, where miR-30a expression in the ductal plate regulates TGF-β, essential for the normal differentiation of hepatoblasts into cholangiocytes and hepatocytes. Failure of ductal plate morphogenesis is associated with approximately 25 % of cases of biliary atresia [24]. Our experiments showed relatively high levels of miR-30a in all liver samples, while miR-30c and 30b were expressed at much lower levels (from 4-fold to 26-fold lower; see Table 5).

#### Mir-23b cluster

Rogler *et al*. (2009) [25] showed that bile duct formation in foetal murine hepatocytes was repressed by high levels of miR-23b, miR-27b and miR-24-1. By downregulating Smad (in particular Smad 4) expression and consequently TGF-β signalling, bile duct gene expression was reduced and hepatocyte growth promoted. Conversely, low levels of miR-23b in cholangiocytes permitted TGF-β signalling and bile duct development. We found low levels of miR-23b and 24-1, consistent with the development of bile ducts, but high levels of miR-27b, suggesting the suppression of bile duct gene expression and a shift toward hepatocyte differentiation.

### Evidence of miRNA expression in response to infection

There are a number of studies investigating the relationship between infection and host miRNA response in liver, mainly focusing on CMV, hepatitis B virus and hepatitis C virus, e.g. [26–31]. Other hepatotropic viruses have been less well studied in this respect, but there have been limited reports on rhesus rotavirus in murine BA models [32, 33] and liver injury associated with HIV-1 infections [34].

We found evidence of CMV infection in eight patients using serology and quantitative CMV DNA testing in whole blood, urine and bile samples. It was interesting to find that CMV infection was diagnosed in three infants with CMV-associated BA, as well as three with isolated BA and two with choledochal malformations. Bearing in mind that the true incidence of congenitally acquired CMV infection is not known, but likely to be underdiagnosed, one could speculate whether CMV-associated BA should be seen more frequently given the low incidence of BA. This does not exclude the involvement of CMV in the aetiology of BA, especially as there is clear evidence of CMV infection in our cohort. However, NGS did not detect any CMV or other known viral miRNA sequences, or any specific host miRNAs that differentiated the CMV-associated samples from the congenital forms. It is possible that looking for a CMV-specific immune response, rather than the genome, might be helpful if biliary atresia was due to an infectious ‘hit and run’ pathogenesis.

The upregulation of miR-17, -20, -96, -182 and -185 in response to CMV infection has been described [27, 28, 30]. In our work, miR-96 was not detected and although miR-182-5p was expressed, it was at similar, low levels in all liver samples, although generally higher in bile samples (see Fig. 4, Table 7). MicroRNA-122 is considered to be a general, non-specific marker of liver injury and its levels are regulated in both liver and serum in a number of liver diseases, including hepatitis B and C virus infection and solid tumours [35]. We found miR-122-5p and miR-122-3p expression in all liver samples, except for sample 6 (BASM), likely due to hepatobiliary injury [36, 37], particularly inflammation and fibrosis [38, 39].

### Inflammation and fibrosis

BA is a liver disease characterized by hepatic injury and inflammation, followed by fibrosis and deposition of extracellular matrix (ECM), distorting the normal liver parenchyma and leading to the obliteration of the extrahepatic bile ducts and portal hypertension [40]. Hepatic stellate cells are the main source of ECM and there have been an increasing number of publications investigating both their function and potential as therapeutic targets to reverse hepatic fibrosis, together with the role of miRNAs in regulating their activation [40]. A number of these miRNAs were identified in our experiments; see Tables 5 and 6 for expression in liver and bile samples, respectively, and also the pathway analysis (Fig. 3, Table 4).

### The miR-29 family

In liver tissues, microRNA-29a-3p is the most abundant member of the family, accounting for 70 % of its overall expression [41]. For the seven samples where at least two members of the family were expressed, we found that on average 73 % of expression was accounted for by miR-29a-3p. From the network analysis (Fig. 3), the mir-29 family are predicted to be involved in numerous interactions, particularly the collagen-encoding genes and the DNA methyltransferase 1 gene (DNMT1). High levels of mir-29 have been shown to have anti-fibrotic effects by suppressing collagen 1 A1 mRNA and its protein expression, reducing HSC activation [42]. Wang *et al*. [43], Szabo and Bala [44] and Kriegel *et al*. [45] showed that low levels of mir-29 are associated with an increase in fibrosis in BA patients. Our results showed that miR-29b-3p was expressed at low levels in all samples, except sample 6 (BASM), where no expression was found. MicroRNA-29c-3p expression was also low and absent in three samples; 5 (BASM), 6 (BASM) and 8 (isolated BA).

### miR-483

The cooperative roles of miR-483-3p/-5p in inhibiting liver fibrosis in transgenic mice has been shown by Li *et al* [46]. Their work demonstrated that by acting together, miR-483-5p/-3p target two pro-ﬁbrosis factors; platelet-derived growth factor-b and tissue inhibitor of metalloproteinase 2, which suppress the activation of hepatic stellate cells (HSCs). Recent work by Chen *et al*. [47] supports these findings.

We found low levels of miR-483-5p and higher levels of miR-483-3p (up to 10-fold higher), although the differences between BA types did not reach statistical significance. The relatively low levels of miR-483-5p in our samples suggest that inflammation and subsequent fibrosis due to the unchecked activation of HSCs is possible.

### MiR-181, miR-199 and miR-200

The miRNet pathway analysis (Fig. 3, Table 4) shows miR-181a-5p, 199a-3p, 200a-3p and 200b-3p associating directly with genes involved with collagen synthesis, ECM and mRNA export and stability. MicroRNA-199a, -200a and -200b were shown to be positively correlated with liver fibrosis and it was shown that there were significant differences in expression according to the classification of the fibrosis [48]. We found relatively high levels of identical expression of miR-199a-3p and -199b-3p in all samples. However, miR-199a-5p, miR-200a and miR-200b were less abundant, with a 10–30-fold reduction in expression compared to miR-199a/b-3p; these differences may be due to differences in the grade of fibrosis between patients.

Zheng *et al*. [49] and Yu *et al*. [50] found that HSC activation and the subsequent development of fibrosis is, at least in part, controlled by phosphatase and tensin homologue (PTEN) expression via a cascade of reactions involving miR-181b. It was also shown that miR-181b expression increased more than miR-181a *in vitro* in TGF-β-induced HSC activation [50]. However, our experiments showed up to 30-fold higher levels of miR-181a expression compared with miR-181b. Brockhausen *et al*. [51] also demonstrated that miR-200 showed little or no differential expression during TGF-β-driven EMT, while miR-181a was significantly induced early on in the process. This seems to correlate with the results we found, with low expression of mir-200a/-200b and higher levels of miR-181a, potentially highlighting a microRNA signature of fibrosis in our patients.

All of these publications and their differing results highlight some of the difficulties in making useful comparisons in microRNA expression studies. In our work, the differences in expression patterns could be due to the fact that the liver tissue analysed is likely to contain a mixture of hepatocytes, cholangiocytes and HSCs, from which a very mixed population of microRNAs would have been sampled.

### MicroRNA expression in bile samples

MicroRNAs in bile fluid are likely derived from both circulating exosomes and also from intact epithelial cells desquamated from the bile tract [52]. One of the difficulties in interpreting our data is the origin of the bile fluid, since the disease itself precludes the presence of distinct bile ducts. Even when sampling from patent ducts, unfractionated bile is known to contain cellular fragments, making it difficult to define a unique mircoRNA profile [53]. This is further complicated by the fact that the mechanisms controlling miRNA release into the bile have yet to be determined. However, our results demonstrate consistent and statistically significant differences between liver and bile samples, highlighting their potential value as a source of biomarkers. Overall, there were 110 mircroRNAs statistically significantly differentially expressed between bile and liver samples (*P* value <0.05). Of these, 54 were more highly expressed in bile samples and 56 more highly expressed in liver samples; Fig. 4, Table 7 illustrate these differences.

### MiR-486

One striking difference in microRNA levels was the high and consistent expression of miR-486-5p in bile samples (Fig. 4, S1 and Table S1), particularly in patients 3 (CMV-associated), 6 (BASM) and 19 (type 1 BA). The values ranged from 9.6×10^4^ c.p.m. to 1.1×10^6^ c.p.m. (patients 8 and 19, respectively). MiR-486-5p was expressed in all liver samples, but at lower levels, ranging from 2.7×10^3^ c.p.m. to 1.4×10^4^ c.p.m. (42 and 4, respectively). The average log_10_ FC between all bile and liver samples was 7.48 (*P*=1.25E−21).

MicroRNA-486 is known to be one of the most abundant miRNAs in red blood cells [54] There have also been a number of reports highlighting the association of miR-486 with fibrosis in the lung [55] and kidney [56, 57], although no clear association with liver inflammation and fibrosis has been established yet. The differential pattern of high expression in bile and relatively low levels in liver tissue in our samples is interesting. If the microRNA is meant to protect against inflammation and fibrosis, the low levels in the liver may be a signature of such damage. The high levels in the bile may also be a key stage in the exosomal transport of mir-486-5p in response to fibrotic injury.

### Conclusion

We have presented a comprehensive analysis of the microRNA expression patterns of bile and liver tissue samples from a small number of patients diagnosed with biliary atresia and control samples with CMV-associated BA, choledochal malformations and BASM. While we were unable to use samples from healthy donors, we anticipated a specific miRNA profile that distinguished between the different forms of the disease. This could either present as known or potentially novel miRNAs from pathogens or a definitive host response to infection.

No differential expression patterns were found that would enable the classification of disease types. What we did find was clear evidence of microRNAs associated with hepatocyte and cholangiocyte differentiation, together with fibrosis and inflammation, and statistically significant differences in expression between bile and liver samples. Whether the differential expression of pro- and anti-inflammatory microRNAs in the two sample types evaluated represents a transient shift from export to import remains to be determined. The differences (and similarities) between circulating microRNAs found in other studies and those found in bile samples in this work are of interest and also require further investigation.

There are a number of limitations to our study. As described in the Introduction we did not have access to true non-BA controls. Rather, our aim was to identify microRNA markers associated with BA and determine if there were patterns of expression that could distinguish the isolated, idiopathic BA, from the better characterized BASM, choledochal malformations and CMV-associated forms of the disease. Although the number of samples tested was low, this is a reflection of the disease prevalence across the different forms of the condition required for the analysis and this also meant that true biological replicates were unavailable. A further issue with our results was that we were unable to independently test our findings by PCR. However, our work is supported in some aspects by others and also highlights the difficulties in interpreting the differences in microRNA expression patterns and levels by the various analytical methods, particularly primer and probe sequence variation in expression arrays and comparing array-based methods and NGS-derived data. For example, there are striking differences between reports on the abundance of miR-200 and miR-181a-5p in inflammation and also difficulties in interpreting the many murine models and their relationship to human disease.

This study has revealed a number of potential biomarkers associated with the development of the liver and bile ducts and liver inflammation. We have found a set of miRNAs that appear to indicate damage to the biliary tract, rather than a specific set of indicators for any one type of biliary atresia. Those associated with liver and bile duct development (miR-30 and the miR-23 cluster) and inflammation and fibrosis (miR-29, miR-483, miR-181, miR-199 and miR-200) need to be investigated in more detail using quantitative real-time RT-PCR.

## Supplementary Data

Supplementary material 1
