# Supplementary material for: Using next-generation sequencing of microRNAs to identify host and/or pathogen nucleic acid signatures in samples from children with biliary atresia – a pilot study
Source: Access Microbiol. 2020 Jun 12;2(7):acmi000127. doi: 10.1099/acmi.0.000127 (PMC7497833; doi:10.1099/acmi.0.000127)
Supplement: Supplementary material 1 [file acmi-2-127-s001.pdf]

Supplementary Tables and figures.

miRNA Name	B19 (T1)	B3 (CMV)	B4 (T1)*	B42 (T1)	B5 (BASM)	B6 (BASM)	B8 (T1)	L26 (T1)	L27 (CC)	L39 (CC)	L4 (T1)*	L42 (T1)	L5 (BASM)	L6 (BASM)	L8 (T1)
hsa-let-7a-1,hsa-let-7a-5p	32841	11734	6179	5265	11986	7280	5207	5555	13520	7139	15466	9478	24498	7259	16850
hsa-let-7a-2,hsa-let-7a-5p	32841	11734	6179	5265	11986	7280	5207	5555	13502	7139	15466	9478	24538	7259	16817
hsa-let-7a-3,hsa-let-7a-5p	32850	11746	6179	5265	11986	7280	5207	5555	13520	7139	15466	9489	24538	7259	16918
hsa-let-7b,hsa-let-7b-5p	4611	1180	3500	3803	4883	1365	8994	2453	2537	2401	3822	1786	2762	2577	3181
hsa-let-7c,hsa-let-7c-5p	626	321	3020	3705	3107	1365	5325	1015	2395	2017	1038	922	2362	526	1658
hsa-let-7d,hsa-let-7d-3p	354	476	20	98	148	0	0	169	71	256	253	57	160	368	135
hsa-let-7d,hsa-let-7d-5p	4303	1267	460	488	444	227	473	254	408	240	557	284	721	158	711
hsa-let-7e,hsa-let-7e-5p	91	31	920	293	444	455	1183	282	444	304	354	478	721	105	1083
hsa-let-7f-1,hsa-let-7f-5p	88748	32001	12559	12090	11246	11147	9349	7360	19588	11588	10378	11150	22536	5313	17121
hsa-let-7f-2,hsa-let-7f-5p	89193	32230	12619	12285	11542	11147	9467	7501	20014	11844	10682	11389	22897	5365	17256
hsa-let-7g,hsa-let-7g-5p	13970	7347	2460	2633	1776	910	947	1748	3513	2193	3493	2321	6685	1946	3654
hsa-let-7i,hsa-let-7i-5p	14378	20490	11219	6728	9914	24797	11716	8742	7150	6979	3772	4631	6405	7574	4094
hsa-mir-100,hsa-miR-100-5p	2106	420	3180	2925	1332	227	6272	3243	1260	5890	1595	2913	1401	3524	1455
hsa-mir-101-1,hsa-miR-101-3p	8015	17481	9679	8093	23823	17745	4024	7219	15454	16822	2607	7646	3563	2157	3214
hsa-mir-101-2,hsa-miR-101-3p	8360	17987	9899	8093	24415	17972	4260	7473	16039	17703	2683	7907	3563	2209	3316
hsa-mir-103a-1,hsa-miR-103a-3p	6218	13952	5819	6338	11246	9782	2604	1297	2236	1985	1747	1252	2402	2525	1218
hsa-mir-103a-2,hsa-miR-103a-3p	6218	13952	5819	6338	11246	9782	2604	1297	2236	1985	1747	1252	2402	2525	1218
hsa-mir-106b,hsa-miR-106b-3p	5020	7421	4600	5558	10506	12057	2012	451	408	224	228	250	280	316	271
hsa-mir-106b,hsa-miR-106b-5p	3132	717	380	195	148	227	237	338	106	208	202	114	80	0	34
hsa-mir-107,hsa-miR-107	2088	9139	4939	5655	10802	8190	2249	338	692	800	380	432	600	1683	305
hsa-mir-10a,hsa-miR-10a-5p	12154	4980	41556	95650	47054	21840	56450	91538	59509	73756	14909	45659	14531	33085	8188
hsa-mir-10b,hsa-miR-10b-5p	3422	5994	74952	385427	114233	136272	79645	18527	41358	21256	2152	10968	5844	947	643
hsa-mir-1180,hsa-miR-1180-3p	36	99	80	0	148	682	118	28	0	48	51	0	40	0	135
hsa-mir-122,hsa-miR-122-3p	27	0	20	0	0	0	0	113	586	864	152	637	160	0	203
hsa-mir-122,hsa-miR-122-5p	1207	12	1440	683	592	227	592	7445	24982	19511	25793	15075	47755	18094	30960
hsa-mir-125a,hsa-miR-125a-5p	1071	43	420	293	0	0	237	2284	1792	2817	1544	1775	560	999	1489

hsa-mir-125b-1,hsa-miR-125b-1-3p	9	19	160	0	0	0	0	28	0	16	0	11	40	53	0
hsa-mir-125b-1,hsa-miR-125b-5p	545	37	200	780	592	227	473	649	692	1537	380	535	400	526	338
hsa-mir-125b-2,hsa-miR-125b-2-3p	0	19	460	488	148	0	592	56	195	112	177	80	240	210	203
hsa-mir-125b-2,hsa-miR-125b-5p	545	37	200	780	592	227	473	649	692	1537	380	535	360	526	305
hsa-mir-126,hsa-miR-126-3p	1471	365	240	0	0	227	0	2425	1331	5842	3569	3755	1001	2209	1184
hsa-mir-126,hsa-miR-126-5p	4148	4919	2920	975	444	227	1065	4004	4223	12277	6733	6212	2162	5628	3147
hsa-mir-127,hsa-miR-127-3p	1244	501	4979	1560	1480	1137	4852	2623	1774	2161	9290	4699	3362	3998	4636
hsa-mir-127,hsa-miR-127-5p	0	6	60	98	0	0	0	197	71	80	25	91	0	0	0
hsa-mir-128-1,hsa-miR-128-3p	681	624	80	0	148	0	118	141	35	48	127	46	120	210	68
hsa-mir-128-2,hsa-miR-128-3p	681	612	80	0	148	0	118	113	35	48	76	46	120	210	68
hsa-mir-1307,hsa-miR-1307-3p	36	37	80	0	0	0	118	0	18	64	101	102	240	105	203
hsa-mir-1307,hsa-miR-1307-5p	354	253	1380	878	2368	1365	473	874	461	608	607	922	520	736	643
hsa-mir-130a,hsa-miR-130a-3p	2505	1940	1180	1658	888	682	1302	1072	1011	1585	481	944	360	736	406
hsa-mir-130b,hsa-miR-130b-3p	762	667	80	390	0	455	0	28	53	16	76	34	0	53	0
hsa-mir-132,hsa-miR-132-3p	27	31	40	0	0	227	0	85	53	64	25	57	0	0	68
hsa-mir-133a-1,hsa-miR-133a-3p	0	12	20	98	0	0	118	0	53	80	0	23	0	0	34
hsa-mir-133a-2,hsa-miR-133a-3p	0	12	20	98	0	0	118	0	53	80	0	23	0	0	34
hsa-mir-134,hsa-miR-134-5p	9	6	40	0	0	0	0	85	53	16	177	68	120	105	102
hsa-mir-136,hsa-miR-136-3p	118	80	440	98	148	0	118	1325	905	816	962	2241	721	684	677
hsa-mir-136,hsa-miR-136-5p	127	25	420	98	0	0	355	338	71	112	253	614	200	368	237
hsa-mir-139,hsa-miR-139-5p	18	0	0	0	0	0	118	113	53	208	51	46	80	53	34
hsa-mir-140,hsa-miR-140-3p	890	859	500	683	148	0	237	705	550	864	354	523	280	316	203
hsa-mir-140,hsa-miR-140-5p	36	19	20	0	0	0	0	28	35	16	25	23	0	0	0
hsa-mir-142,hsa-miR-142-3p	526	142	0	0	0	0	0	367	35	128	253	228	40	105	203
hsa-mir-142,hsa-miR-142-5p	24427	17604	2080	2243	444	227	1065	2115	319	1569	2278	2264	440	947	372
hsa-mir-143,hsa-miR-143-3p	3631	2373	57594	56259	25747	3867	39172	104002	76755	99942	45334	42063	67249	57860	45780
hsa-mir-144,hsa-miR-144-3p	32242	8502	4460	878	3255	1137	1183	649	18	256	886	250	40	158	68
hsa-mir-144,hsa-miR-144-5p	33522	2638	400	390	296	682	355	56	35	32	228	34	360	0	102

hsa-mir-145,hsa-miR-145-3p	64	6	60	98	0	0	118	113	53	80	25	34	40	210	0
hsa-mir-145,hsa-miR-145-5p	45	25	140	195	296	227	0	1607	958	1329	354	455	0	316	237
hsa-mir-146a,hsa-miR-146a-5p	463	167	1180	293	148	455	828	451	195	208	709	239	520	526	474
hsa-mir-146b,hsa-miR-146b-3p	54	31	20	195	0	0	0	28	319	224	76	159	360	158	135
hsa-mir-146b,hsa-miR-146b-5p	1561	222	2280	975	148	1820	1893	7022	6760	1697	6733	4892	10808	7469	2944
hsa-mir-148a,hsa-miR-148a-3p	2415	8669	12919	43974	5475	1592	13373	24224	84367	129105	10175	47502	16332	14044	7241
hsa-mir-148a,hsa-miR-148a-5p	45	37	160	975	444	0	118	28	727	416	152	102	160	53	237
hsa-mir-148b,hsa-miR-148b-3p	54	365	100	0	0	0	237	705	426	1329	278	853	120	210	237
hsa-mir-149,hsa-miR-149-5p	0	6	20	195	148	0	0	0	18	48	0	11	0	0	102
hsa-mir-150,hsa-miR-150-5p	454	420	60	390	0	2275	355	85	18	32	177	137	0	0	102
hsa-mir-151a,hsa-miR-151a-3p	2732	3615	1120	1463	740	227	473	1213	1011	1969	1266	1081	1121	1315	880
hsa-mir-151a,hsa-miR-151a-5p	10920	2268	1220	585	1480	682	1775	423	674	688	1493	592	1001	1210	1793
hsa-mir-151b,hsa-miR-151b	790	389	160	98	296	227	0	28	18	48	76	0	120	53	68
hsa-mir-152,hsa-miR-152-3p	54	0	40	98	148	0	0	113	124	208	0	148	0	0	68
hsa-mir-154,hsa-miR-154-5p	0	0	20	0	0	0	0	28	35	48	25	46	0	53	34
hsa-mir-155,hsa-miR-155-5p	36	56	60	0	0	0	118	141	0	32	202	91	120	0	0
hsa-mir-15a,hsa-miR-15a-5p	18136	11530	2280	975	2368	1137	118	338	248	240	127	205	80	316	169
hsa-mir-15b,hsa-miR-15b-3p	590	136	40	0	148	0	0	28	0	64	0	34	0	0	0
hsa-mir-15b,hsa-miR-15b-5p	4747	797	220	195	0	0	118	113	71	128	127	102	120	53	34
hsa-mir-16-1,hsa-miR-16-5p	277989	107924	9619	8093	1776	1592	2840	2707	958	1857	3367	1957	1161	3261	1692
hsa-mir-16-2,hsa-miR-16-2-3p	6626	2367	560	293	740	455	0	56	0	0	25	46	0	0	0
hsa-mir-16-2,hsa-miR-16-5p	278098	107961	9639	8093	1776	1592	2840	2707	940	1841	3367	1946	1201	3261	1692
hsa-mir-17,hsa-miR-17-3p	599	148	60	0	148	0	0	0	35	16	0	0	40	53	34
hsa-mir-17,hsa-miR-17-5p	1461	377	80	488	0	0	0	113	284	448	228	341	160	105	237
hsa-mir-181a-1,hsa-miR-181a-3p	27	31	140	98	296	455	0	310	89	112	127	80	200	158	68
hsa-mir-181a-1,hsa-miR-181a-5p	37643	32842	34576	60744	46906	87587	47574	11675	18240	11348	23388	10820	28381	34716	26257
hsa-mir-181a-2,hsa-miR-181a-2-3p	45	37	120	195	148	0	118	141	319	320	127	228	80	105	271
hsa-mir-181a-2,hsa-miR-181a-5p	37643	32842	34576	60744	46906	87587	47574	11675	18240	11348	23388	10820	28381	34716	26257

hsa-mir-181b-1,hsa-miR-181b-5p	1062	630	1520	1268	1924	7280	2367	677	674	704	886	455	841	999	1523
hsa-mir-181b-2,hsa-miR-181b-5p	1080	630	1540	1268	1924	7280	2367	761	727	752	886	455	841	999	1523
hsa-mir-181c,hsa-miR-181c-3p	18	6	20	0	0	0	118	28	18	16	51	11	0	0	68
hsa-mir-181c,hsa-miR-181c-5p	327	99	780	293	592	227	947	620	426	512	304	603	400	789	440
hsa-mir-181d,hsa-miR-181d-5p	18	19	100	98	148	0	0	113	35	48	25	57	40	0	0
hsa-mir-182,hsa-miR-182-5p	24055	18352	4540	3510	888	910	0	959	319	272	481	569	280	579	541
hsa-mir-183,hsa-miR-183-5p	1452	1248	180	98	0	0	118	85	0	80	127	91	80	0	0
hsa-mir-185,hsa-miR-185-5p	536	31	20	0	296	0	118	56	18	48	0	11	40	0	0
hsa-mir-186,hsa-miR-186-5p	8596	8657	5939	2243	3699	3867	2959	1410	798	2561	1671	1570	921	2893	575
hsa-mir-18a,hsa-miR-18a-5p	154	43	40	0	0	0	0	592	195	944	127	922	0	0	0
hsa-mir-18b,hsa-miR-18b-5p	9	6	20	0	0	0	0	28	18	32	0	11	0	0	0
hsa-mir-191,hsa-miR-191-5p	17301	20768	4380	10043	1184	2957	3550	2735	4223	3809	5037	4301	4163	2157	2267
hsa-mir-192,hsa-miR-192-5p	34984	31068	54674	26813	15093	8190	8166	27862	74342	114891	55813	53657	127693	42764	50348
hsa-mir-193b,hsa-miR-193b-3p	36	6	80	195	0	0	0	197	266	432	76	171	80	0	68
hsa-mir-194-1,hsa-miR-194-5p	1815	1372	100	0	0	0	0	338	781	1152	937	683	520	1052	947
hsa-mir-194-2,hsa-miR-194-5p	1979	1434	100	0	0	0	0	451	1047	1377	1088	842	640	1420	1184
hsa-mir-195,hsa-miR-195-5p	91	6	40	98	0	0	118	310	160	352	101	148	0	0	0
hsa-mir-197,hsa-miR-197-3p	64	19	20	98	148	0	0	254	106	128	76	91	80	53	135
hsa-mir-199a-1,hsa-miR-199a-3p	5782	1551	12059	10140	8582	455	16450	8206	2661	5986	3974	4722	2882	4629	3823
hsa-mir-199a-1,hsa-miR-199a-5p	726	105	680	585	0	0	710	1325	621	1184	759	978	200	631	744
hsa-mir-199a-2,hsa-miR-199a-3p	5782	1551	12059	10140	8582	455	16450	8206	2661	5986	3974	4722	2882	4629	3823
hsa-mir-199a-2,hsa-miR-199a-5p	726	105	680	585	0	0	710	1325	621	1184	759	978	200	631	744
hsa-mir-199b,hsa-miR-199b-3p	5782	1551	12059	10140	8582	455	16450	8206	2661	5986	3974	4722	2882	4629	3823
hsa-mir-199b,hsa-miR-199b-5p	454	62	400	195	148	0	1183	508	195	176	101	262	40	368	169
hsa-mir-19a,hsa-miR-19a-3p	917	439	240	98	148	227	0	1213	834	1777	51	740	40	53	0
hsa-mir-19b-1,hsa-miR-19b-3p	2669	1576	1060	195	296	910	118	5443	3655	7555	456	2469	200	263	237
hsa-mir-19b-2,hsa-miR-19b-3p	2678	1576	1060	195	296	910	118	5443	3655	7571	456	2458	200	263	237
hsa-mir-200a,hsa-miR-200a-3p	200	37	20	98	0	0	0	479	248	272	127	262	80	368	474
hsa-mir-200b,hsa-miR-200b-3p	172	6	20	0	0	0	0	310	18	208	177	102	40	263	203

hsa-mir-203a,hsa-miR-203a	18	0	20	0	0	0	118	85	89	96	76	68	40	0	68
hsa-mir-204,hsa-miR-204-5p	136	62	40	390	148	0	237	874	639	976	228	171	80	158	68
hsa-mir-20a,hsa-miR-20a-5p	681	185	120	0	296	0	0	282	515	720	304	546	200	105	237
hsa-mir-21,hsa-miR-21-3p	0	37	500	1170	148	0	355	5668	3424	5426	2683	4050	5124	3261	1286
hsa-mir-21,hsa-miR-21-5p	24926	13853	18318	15893	8878	1592	7811	54201	22924	26010	15238	38229	20455	22197	12587
hsa-mir-210,hsa-miR-210-3p	708	488	300	0	296	1820	0	85	0	32	25	68	0	53	34
hsa-mir-214,hsa-miR-214-3p	163	25	100	0	296	0	237	197	231	224	101	137	40	53	102
hsa-mir-214,hsa-miR-214-5p	100	0	60	0	0	0	0	56	18	80	76	23	0	53	34
hsa-mir-218-1,hsa-miR-218-5p	36	49	80	195	148	0	118	56	53	32	25	57	40	105	0
hsa-mir-218-2,hsa-miR-218-5p	36	49	80	195	148	0	118	56	53	32	25	46	40	105	0
hsa-mir-22,hsa-miR-22-3p	30372	37686	64373	11993	8878	31395	28639	19938	12136	16726	29311	17260	16732	34926	21757
hsa-mir-221,hsa-miR-221-3p	363	260	440	98	148	0	355	451	266	464	405	444	240	316	508
hsa-mir-222,hsa-miR-222-3p	136	43	80	0	148	0	355	56	18	160	278	182	120	158	338
hsa-mir-223,hsa-miR-223-3p	753	136	120	0	296	0	0	169	18	240	430	375	120	158	102
hsa-mir-224,hsa-miR-224-5p	36	12	20	0	0	0	0	0	0	16	25	0	40	53	0
hsa-mir-23a,hsa-miR-23a-3p	808	241	360	390	296	455	355	451	248	416	202	273	80	263	237
hsa-mir-23b,hsa-miR-23b-3p	545	136	140	195	0	227	355	451	1065	848	658	466	280	684	237
hsa-mir-24-1,hsa-miR-24-3p	272	43	200	0	0	227	237	451	213	656	304	353	320	736	406
hsa-mir-24-2,hsa-miR-24-3p	272	43	200	0	0	227	237	451	213	656	304	353	320	736	406
hsa-mir-25,hsa-miR-25-3p	16784	40003	6759	12480	4735	16607	2130	3017	3300	3505	2025	2321	2082	2209	1286
hsa-mir-26a-1,hsa-miR-26a-5p	37353	9880	6019	3120	5179	1365	10059	37309	31121	56725	22224	33018	13090	42974	12350
hsa-mir-26a-2,hsa-miR-26a-5p	37362	9880	6019	3120	5179	1365	10059	37309	31121	56725	22224	33018	13090	42974	12350
hsa-mir-26b,hsa-miR-26b-3p	18	31	0	0	0	0	118	56	71	64	0	34	0	158	0
hsa-mir-26b,hsa-miR-26b-5p	9912	4659	2320	975	1036	1137	2012	1946	5039	5826	3063	5074	2202	2051	2335
hsa-mir-27a,hsa-miR-27a-3p	2469	290	980	293	296	227	118	1213	568	1184	962	785	761	1631	1150
hsa-mir-27a,hsa-miR-27a-5p	9	6	40	98	0	0	0	197	0	80	25	46	40	53	0
hsa-mir-27b,hsa-miR-27b-3p	10756	9676	43116	11895	3995	1820	13254	19007	22498	39487	37057	19012	46794	61226	42025
hsa-mir-27b,hsa-miR-27b-5p	18	0	20	0	0	0	0	0	53	32	25	23	40	105	102
hsa-mir-28,hsa-miR-28-3p	436	383	2700	0	740	0	2130	1579	1828	2481	1367	1536	1401	1894	1692

hsa-mir-299,hsa-miR-299-3p	9	19	80	0	0	0	237	197	71	80	152	148	40	0	68
hsa-mir-29a,hsa-miR-29a-3p	808	111	260	293	0	0	0	1213	639	1489	835	740	160	473	338
hsa-mir-29b-1,hsa-miR-29b-3p	91	31	0	0	0	0	0	169	124	368	51	102	80	0	34
hsa-mir-29b-2,hsa-miR-29b-3p	91	31	0	0	0	0	0	169	124	368	51	114	80	0	34
hsa-mir-29c,hsa-miR-29c-3p	309	117	0	98	0	0	0	85	71	192	51	91	0	0	0
hsa-mir-301a,hsa-miR-301a-3p	554	519	140	0	148	0	118	56	124	80	51	137	0	53	34
hsa-mir-30a,hsa-miR-30a-3p	45	6	280	98	0	0	237	338	160	448	101	193	0	53	102
hsa-mir-30a,hsa-miR-30a-5p	926	303	4320	1755	296	682	2604	8065	8534	7971	6353	5439	6965	10941	4399
hsa-mir-30b,hsa-miR-30b-5p	672	606	60	98	148	0	0	564	1136	832	582	569	600	421	778
hsa-mir-30c-1,hsa-miR-30c-5p	2106	741	80	0	0	0	355	649	763	1072	658	523	881	421	1083
hsa-mir-30c-2,hsa-miR-30c-2-3p	0	0	20	0	0	0	237	28	35	16	25	11	0	0	0
hsa-mir-30c-2,hsa-miR-30c-5p	2106	741	80	0	0	0	355	649	763	1072	658	523	881	421	1083
hsa-mir-30d,hsa-miR-30d-3p	27	49	60	0	148	0	0	0	18	32	51	0	0	0	34
hsa-mir-30d,hsa-miR-30d-5p	4221	3745	2620	1755	1184	2957	1065	3243	3815	5282	6227	2822	6685	8679	6429
hsa-mir-30e,hsa-miR-30e-3p	191	130	440	0	0	0	710	56	426	624	228	239	240	105	169
hsa-mir-30e,hsa-miR-30e-5p	3059	2552	940	293	444	0	237	3187	2378	4370	5670	3072	2882	6522	3316
hsa-mir-32,hsa-miR-32-5p	753	290	160	98	148	0	0	197	106	320	51	102	0	105	237
hsa-mir-320a,hsa-miR-320a	1089	2299	2780	2925	3699	7962	1657	423	319	368	304	193	320	368	237
hsa-mir-320b-1,hsa-miR-320b	91	260	920	390	740	2275	237	56	35	48	25	23	0	105	0
hsa-mir-320b-2,hsa-miR-320b	91	260	920	390	740	2275	237	56	35	48	25	23	0	105	0
hsa-mir-323a,hsa-miR-323a-3p	18	25	80	98	0	227	0	0	53	32	76	91	120	53	135
hsa-mir-324,hsa-miR-324-5p	182	31	0	0	0	455	0	28	18	16	51	137	0	0	0
hsa-mir-331,hsa-miR-331-3p	18	6	0	0	0	0	0	85	35	80	25	57	40	53	34
hsa-mir-335,hsa-miR-335-3p	54	12	20	0	0	0	0	197	89	240	25	102	40	210	102
hsa-mir-335,hsa-miR-335-5p	109	56	60	98	148	0	237	169	53	80	101	46	0	53	34
hsa-mir-337,hsa-miR-337-3p	18	0	0	0	0	0	0	85	0	16	25	34	40	0	34
hsa-mir-337,hsa-miR-337-5p	0	6	20	0	0	0	0	28	0	16	25	34	80	53	34
hsa-mir-338,hsa-miR-338-3p	73	6	120	0	0	0	0	56	18	16	25	0	40	53	68
hsa-mir-339,hsa-miR-339-3p	64	62	100	0	0	0	118	0	35	16	76	0	0	0	0

hsa-mir-339,hsa-miR-339-5p	36	105	40	0	0	0	0	85	18	80	25	34	0	105	0
hsa-mir-33b,hsa-miR-33b-5p	345	290	180	0	148	0	0	28	0	48	76	11	80	53	68
hsa-mir-340,hsa-miR-340-5p	227	346	540	683	296	227	0	931	1189	1136	51	410	240	158	135
hsa-mir-342,hsa-miR-342-3p	127	56	120	195	0	682	118	790	124	208	177	80	120	0	0
hsa-mir-345,hsa-miR-345-5p	245	142	200	98	0	682	0	85	35	80	127	68	40	0	271
hsa-mir-34a,hsa-miR-34a-5p	45	0	180	0	0	0	118	338	53	16	152	193	160	105	203
hsa-mir-361,hsa-miR-361-3p	45	12	60	98	148	0	0	56	71	64	76	102	80	263	68
hsa-mir-361,hsa-miR-361-5p	64	31	40	195	0	0	0	28	213	144	278	80	80	105	68
hsa-mir-3613,hsa-miR-3613-5p	27	25	0	0	0	0	0	0	35	96	25	57	0	0	34
hsa-mir-3615,hsa-miR-3615	300	297	120	98	0	910	0	0	0	16	25	0	40	0	34
hsa-mir-362,hsa-miR-362-5p	18	0	20	0	0	0	0	28	35	64	0	57	40	0	0
hsa-mir-363,hsa-miR-363-3p	2932	3108	520	390	148	682	237	0	18	32	101	23	0	53	0
hsa-mir-369,hsa-miR-369-3p	18	43	120	0	148	0	0	0	35	32	177	228	0	158	68
hsa-mir-369,hsa-miR-369-5p	45	6	100	0	0	0	0	0	0	16	51	23	40	105	102
hsa-mir-370,hsa-miR-370-3p	9	0	0	0	0	0	118	0	35	0	152	11	40	53	68
hsa-mir-374a,hsa-miR-374a-3p	281	93	40	0	148	0	0	169	142	288	127	262	240	263	34
hsa-mir-374a,hsa-miR-374a-5p	1107	229	0	0	0	0	0	169	71	144	51	46	0	53	34
hsa-mir-374b,hsa-miR-374b-5p	327	167	0	0	0	227	118	113	89	224	25	114	80	53	68
hsa-mir-375,hsa-miR-375	27	25	160	0	0	0	0	169	426	1264	76	205	400	210	474
hsa-mir-376a-1,hsa-miR-376a-5p	0	0	40	0	148	227	0	56	35	16	51	46	0	0	68
hsa-mir-376b,hsa-miR-376b-3p	9	6	0	0	0	0	118	28	18	0	0	11	0	0	68
hsa-mir-376c,hsa-miR-376c-3p	0	0	20	0	0	0	0	141	53	144	25	80	0	0	34
hsa-mir-378a,hsa-miR-378a-3p	790	1427	2800	2730	3551	3867	1302	2341	3992	2577	658	1934	721	1736	440
hsa-mir-378c,hsa-miR-378c	54	117	260	0	148	227	0	169	177	96	25	125	40	158	34
hsa-mir-379,hsa-miR-379-5p	0	0	0	0	0	0	0	85	53	32	51	46	80	0	34
hsa-mir-381,hsa-miR-381-3p	54	142	600	2048	444	682	473	677	1668	416	810	1001	1081	1052	372
hsa-mir-409,hsa-miR-409-3p	154	130	680	1170	444	0	592	959	852	144	861	796	640	368	812
hsa-mir-410,hsa-miR-410-3p	9	173	400	585	444	0	0	338	550	224	557	626	921	368	744
hsa-mir-411,hsa-miR-411-5p	191	124	200	195	148	227	237	197	550	336	2405	1070	2722	579	1895

hsa-mir-421,hsa-miR-421	236	408	220	0	0	0	237	28	35	32	51	68	40	53	34
hsa-mir-423,hsa-miR-423-3p	418	494	800	878	1036	3185	473	479	266	288	506	228	480	316	947
hsa-mir-423,hsa-miR-423-5p	10439	19056	16218	10433	20568	32760	11361	56	177	112	228	114	320	105	981
hsa-mir-424,hsa-miR-424-3p	218	260	300	195	444	455	237	56	0	16	25	57	80	53	34
hsa-mir-424,hsa-miR-424-5p	281	37	200	98	0	0	355	536	444	1553	481	1297	40	1105	68
hsa-mir-425,hsa-miR-425-3p	64	31	20	0	0	0	0	28	35	48	51	23	0	158	34
hsa-mir-425,hsa-miR-425-5p	2732	2268	260	98	0	227	0	169	106	208	228	137	120	158	203
hsa-mir-429,hsa-miR-429	45	6	60	0	0	0	0	169	35	128	0	91	0	53	102
hsa-mir-431,hsa-miR-431-3p	0	6	60	0	0	0	0	0	53	0	51	46	40	0	68
hsa-mir-431,hsa-miR-431-5p	0	6	40	0	148	0	118	28	18	0	152	114	240	105	169
hsa-mir-432,hsa-miR-432-5p	9	43	340	488	444	0	118	197	124	32	228	114	160	210	338
hsa-mir-4485,hsa-miR-4485	9	25	20	0	0	0	473	28	53	144	152	137	240	0	203
hsa-mir-450a-1,hsa-miR-450a-5p	54	6	60	0	0	0	118	169	18	80	51	68	0	53	34
hsa-mir-450a-2,hsa-miR-450a-5p	54	6	60	0	0	0	118	169	18	80	51	68	0	53	34
hsa-mir-450b,hsa-miR-450b-5p	36	62	100	0	0	0	0	169	18	48	76	68	40	158	135
hsa-mir-451a,hsa-miR-451a	300319	56063	34196	47971	50754	18882	9231	18020	5270	23369	5037	7327	640	1157	474
hsa-mir-455,hsa-miR-455-3p	0	0	0	0	0	0	0	28	106	32	51	23	40	0	102
hsa-mir-455,hsa-miR-455-5p	45	0	80	0	0	0	118	226	444	528	177	307	120	53	34
hsa-mir-4677,hsa-miR-4677-3p	9	19	0	0	0	0	0	0	53	32	25	11	40	0	0
hsa-mir-483,hsa-miR-483-3p	45	6	80	98	0	227	118	2171	3744	5666	1266	1593	1721	789	1895
hsa-mir-483,hsa-miR-483-5p	54	0	160	0	296	0	0	141	124	240	152	68	160	210	305
hsa-mir-484,hsa-miR-484	1879	766	40	390	296	682	355	113	160	208	152	137	80	53	271
hsa-mir-485,hsa-miR-485-3p	0	0	20	0	0	0	0	85	0	32	127	57	0	105	34
hsa-mir-486,hsa-miR-486-5p	1125918	1692948	223877	412533	442282	961869	96213	10688	3797	9011	14149	2651	9767	6154	2775
hsa-mir-487b,hsa-miR-487b-3p	18	12	20	0	0	0	0	113	35	16	228	171	160	53	102
hsa-mir-493,hsa-miR-493-3p	27	12	120	0	148	0	118	28	0	0	202	57	40	53	68
hsa-mir-493,hsa-miR-493-5p	82	37	80	195	0	0	0	85	89	48	152	182	240	158	271
hsa-mir-497,hsa-miR-497-5p	45	43	100	98	296	0	118	28	18	112	76	57	0	0	102
hsa-mir-500a,hsa-miR-500a-3p	91	111	100	98	148	227	237	56	0	32	202	57	240	158	135

hsa-mir-501,hsa-miR-501-3p	82	87	120	0	0	0	237	0	18	48	329	91	40	210	34
hsa-mir-502,hsa-miR-502-3p	64	43	0	0	148	0	0	56	0	16	51	0	40	0	68
hsa-mir-503,hsa-miR-503-5p	18	12	20	0	0	0	0	28	0	16	0	34	0	53	0
hsa-mir-505,hsa-miR-505-3p	9	25	0	0	0	0	0	85	35	32	0	23	0	53	0
hsa-mir-5096,hsa-miR-5096	0	6	20	0	0	227	118	0	0	0	0	0	40	53	68
hsa-mir-532,hsa-miR-532-5p	200	779	700	1073	296	2047	592	310	231	464	152	444	320	421	135
hsa-mir-542,hsa-miR-542-3p	36	12	20	0	148	0	118	282	337	304	202	205	40	210	102
hsa-mir-574,hsa-miR-574-3p	182	19	40	390	0	0	118	592	1153	1665	253	683	40	947	169
hsa-mir-574,hsa-miR-574-5p	45	25	240	0	148	682	118	0	35	0	152	46	40	0	68
hsa-mir-582,hsa-miR-582-3p	9	0	0	0	0	0	0	254	426	368	127	125	40	0	0
hsa-mir-584,hsa-miR-584-5p	254	945	60	0	444	0	0	0	0	0	25	0	80	53	0
hsa-mir-589,hsa-miR-589-5p	209	111	120	0	0	0	0	28	35	16	25	0	0	0	34
hsa-mir-598,hsa-miR-598-3p	27	37	100	98	0	227	0	85	89	128	0	34	0	105	0
hsa-mir-652,hsa-miR-652-3p	536	408	140	98	0	0	237	28	0	16	25	46	40	0	34
hsa-mir-654,hsa-miR-654-3p	163	49	220	780	0	227	0	592	444	336	633	660	200	105	541
hsa-mir-660,hsa-miR-660-5p	381	618	980	878	1332	682	473	536	355	784	76	592	40	158	68
hsa-mir-675,hsa-miR-675-3p	18	0	20	0	0	0	0	85	53	144	51	46	40	53	34
hsa-mir-744,hsa-miR-744-5p	209	210	220	195	148	0	118	0	53	16	76	11	40	53	102
hsa-mir-769,hsa-miR-769-5p	91	87	720	585	740	455	473	451	213	288	304	216	320	421	305
hsa-mir-7704,hsa-miR-7704	27	6	20	0	0	455	0	0	53	32	0	0	40	53	34
hsa-mir-7706,hsa-miR-7706	73	111	60	293	148	0	118	0	18	0	25	0	40	0	34
hsa-mir-874,hsa-miR-874-3p	9	19	40	0	0	0	237	0	53	16	25	11	40	0	34
hsa-mir-877,hsa-miR-877-5p	36	43	100	0	0	455	237	0	0	16	25	0	0	0	0
hsa-mir-885,hsa-miR-885-5p	9	12	40	98	0	0	0	254	337	448	101	80	80	158	169
hsa-mir-889,hsa-miR-889-3p	36	105	340	683	444	0	118	310	195	80	304	478	320	421	68
hsa-mir-92a-1,hsa-miR-92a-3p	6054	41480	19698	25936	22787	16835	6982	5640	10078	13237	13719	7816	10928	9363	14786
hsa-mir-92a-2,hsa-miR-92a-3p	5537	38662	18978	24766	22343	15470	7219	4935	8871	11796	12428	6872	9967	8521	14245
hsa-mir-92b,hsa-miR-92b-3p	354	513	120	195	296	0	0	141	18	48	152	91	160	105	440
hsa-mir-93,hsa-miR-93-5p	6908	7681	1320	1268	592	1365	118	479	727	832	557	410	280	316	305

hsa-mir-941-1,hsa-miR-941	263	62	20	0	0	0	0	56	35	48	228	80	80	53	102
hsa-mir-941-2,hsa-miR-941	263	62	20	0	0	0	0	56	35	48	228	80	80	53	102
hsa-mir-941-3,hsa-miR-941	263	62	20	0	0	0	0	56	35	48	228	80	80	53	102
hsa-mir-941-4,hsa-miR-941	263	62	20	0	0	0	0	56	35	48	228	80	80	53	102
hsa-mir-98,hsa-miR-98-5p	1380	426	240	293	296	455	118	2594	2679	3681	1645	2685	2482	2051	1252
hsa-mir-99a,hsa-miR-99a-3p	0	12	20	0	148	0	118	56	53	112	0	46	0	0	0
hsa-mir-99a,hsa-miR-99a-5p	254	142	720	488	296	0	2130	367	355	944	456	432	240	473	541
hsa-mir-99b,hsa-miR-99b-3p	9	0	0	98	0	0	118	56	89	48	25	34	40	53	0
hsa-mir-99b,hsa-miR-99b-5p	3068	488	2740	2340	1628	227	5562	5612	1313	6835	3620	5939	1561	6680	3011

Table S1. The normalised CPM for the 265 microRNAs identified from the 7 bile and 8 liver samples in the study. MicroRNAs with identical fold-change patterns, but derived from different genetic loci have been retained. B = bile; L = liver; T1 or isolated BA; CC= choledochal malformation; BASM = biliary splenic malformation; CMV = CMV-associated BA. *B4 and L4 were from a patient originally classified as isolated BA, but CMV-positive on testing, see Table 2.

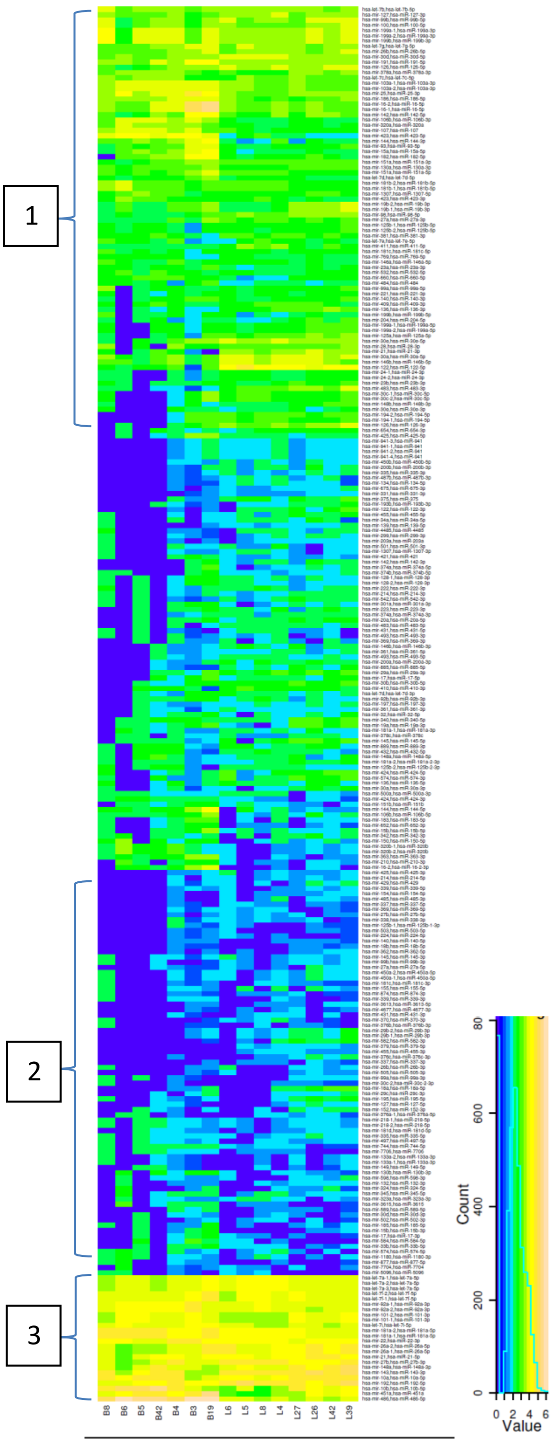

Fig. S1. Heat map showing the expression levels of all 265 microRNAs. B= bile; L= liver. The map was constructed using the data shown in Table 7. Blue represents low expression and orange high expression. The expression patterns are broadly divided into: 1 intermediate; 2 low and 3 high.
